# Lysine Propionylation as a Metabolically Coupled PTM: Mechanisms, Functional Consequences, and Therapeutic Potentials

**DOI:** 10.3390/ijms27072937

**Published:** 2026-03-24

**Authors:** Zhuofan Liu, Xiaoqiang Wang, Lin Li

**Affiliations:** School of Basic Medicine, Faculty of Medicine, Dalian University of Technology, Dalian 116024, China; liuzhuofan923@dlut.edu.cn (Z.L.);

**Keywords:** lysine propionylation (Kpr), lysine acylation, propionyl-CoA, epigenetic regulation, chromatin remodeling, histone modification, metabolic reprogramming

## Abstract

Lysine propionylation (Kpr) is a metabolically coupled lysine acylation that links propionyl-CoA availability to the molecular regulation of gene expression and protein function. Although lysine acetylation (Kac) is the most extensively characterized, recent proteomic and metabolic studies suggest that Kpr is more frequent than previously appreciated, occurs at defined lysine sites, and displays tissue-resolved and context-dependent patterns. Kpr often co-varies with other short-chain acylations such as Kac and lysine butyrylation (Kbu); however, emerging genomic-scale evidence indicates mark-biased genomic distributions and functional associations, suggesting that Kpr is not simply an extension or alternative to Kac. Notably, propionyl-CoA, the direct acyl donor for Kpr, can be influenced by microbiome-derived short-chain fatty acids (SCFAs), implying that interventions modulating SCFA availability (e.g., dietary manipulation) may provide an actionable route to tune Kpr and related acylations. Here, we summarize recent advances in propionyl-CoA sources and compartmentalization, the enzymatic writers/erasers/readers, the molecular mechanisms underlying Kpr, and the functional consequences of Kpr in physiology and disease.

## 1. Introduction

Post-translational modifications (PTMs) play central roles in regulating gene expression and protein function. Lysine acylation refers to the covalent addition of an acyl group to the lysine ε-amino group and represents a major class of metabolite-responsive PTMs. To date, other than lysine acetylation, multiple lysine acylations have been reported, including propionylation, butyrylation, crotonylation, malonylation, succinylation, lactylation, and 2-hydroxyisobutyrylation [1]. These marks are coupled to cellular metabolism through their dependence on acyl-CoA donors, thereby providing a mechanistic interface between metabolic state and epigenetic regulation. By incorporating fluctuations in acyl-CoA pools, driven by nutrient metabolism, hypoxia, inflammation, and microbial activity into histones and non-histone proteins, lysine acylations can modulate protein function, chromatin accessibility, and transcriptional programs, establishing tunable nodes of metabolic-epigenetic coupling in health and disease [1,2,3].

Among lysine acylations, lysine acetylation (Kac) has been studied for the longest time and remains the best characterized. Lysine propionylation (Kpr) differs from Kac by a single additional methylene group and involves the addition of a three-carbon propionyl moiety to lysine residues. The subtle structural change alters side-chain sterics and hydrophobicity and may affect local interaction networks at protein-DNA and protein-protein interfaces [4,5]. However, because Kpr and Kac are closely related, it has been challenging to disentangle whether Kpr primarily mirrors Kac or can exert mark-biased regulatory effects under specific metabolic or pathological contexts.

**Figure 1:**
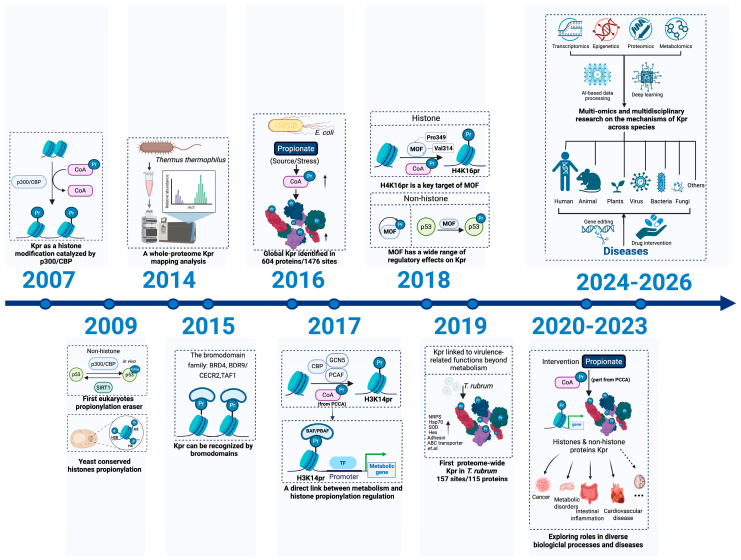
Timeline of lysine propionylation research. Timeline summarizing key advances in the identification, detection, and functional characterization of Kpr. The field began with the discovery of Kpr as a p300/CBP-catalyzed histone modification (2007), followed by the identification of depropionylases and conserved histone Kpr sites (2009), and subsequent expansion of proteome-wide Kpr mapping across organisms and physiological states enabled by improved mass spectrometry workflows (2014–2016). Later studies linked propionyl-CoA availability to site-selective histone propionylation and reader engagement (e.g., bromodomain/BAF–PBAF-associated recognition) (2015–2017), and established enzymatic control of prominent Kpr sites such as H4K16pr via MOF/KAT8 (2018). Proteome-scale datasets in fungi extended Kpr profiling to pathogenic organisms and highlighted Kpr beyond core metabolic proteins (2019). From 2020 onward, metabolic perturbation and disease-oriented studies, together with emerging multi-omics and computational approaches, have accelerated efforts to resolve context-dependent Kpr regulation across tissues and disease settings (2020–2026). Created in BioRender. Z.L. (2026) https://BioRender.com.

Over the past several years, improved proteomic workflows and quantitative metabolite measurements have expanded the catalog of endogenous Kpr sites and clarified that Kpr is dynamically regulated by metabolic inputs [6,7]. Recent evidence indicates that while these short-chain lysine acylations can function in a concerted manner, they can also display context-dependent preferences in genomic distribution and lead to distinct functional consequences [8,9] (Figure 1).

### 1.1. Sources and Metabolic Diversion of Propionyl-CoA

**Figure 2:**
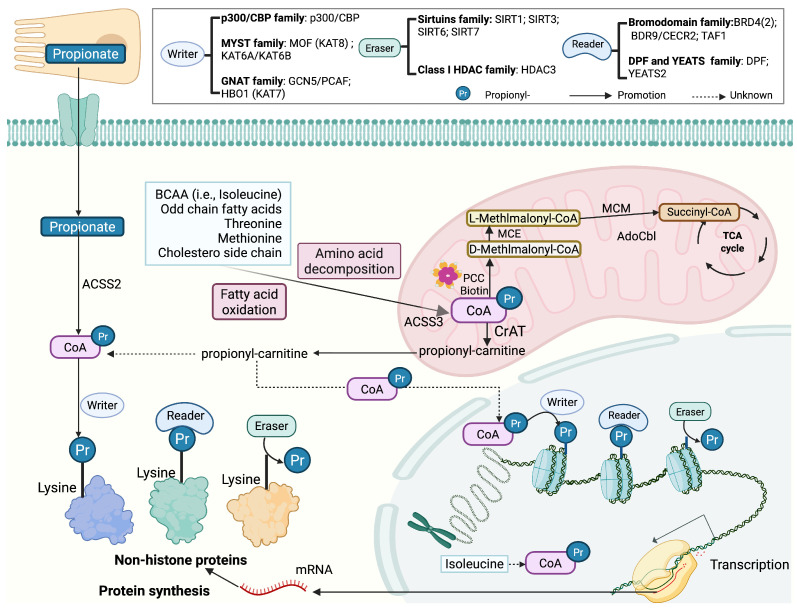
Metabolic origins, compartmentalization, and regulatory network of propionyl-CoA and Kpr. This schematic summarizes major sources of propionyl-CoA and the writer/eraser/reader network governing Kpr on histone and non-histone proteins. Exogenous propionate (e.g., microbiota-derived) can be activated to propionyl-CoA by acyl-CoA synthetases (e.g., ACSS2), whereas endogenous propionyl-CoA is generated primarily in mitochondria through catabolism of propiogenic substrates. Mitochondrial propionyl-CoA is normally routed to the TCA cycle via the propionyl-CoA carboxylase/methylmalonyl-CoA/succinyl-CoA pathway. When propionyl-CoA accumulates, a fraction can be buffered as propionyl-carnitine and redistributed to extra-mitochondrial compartments. Propionyl-CoA can also be produced in nucleus via catabolism of BCAA (e.g., isoleucine). Propionyl-CoA serves as the direct acyl donor for Kpr, which is written by KATs, erased by sirtuins and class I HDACs, and recognized by multiple reader modules. Kpr influences chromatin organization and transcriptional activity, whereas non-histone propionylation modulates protein stability and synthesis. Created in BioRender. Z.L. (2026) https://BioRender.com.

As shown in Figure 2, propionyl-CoA is the immediate acyl donor for lysine propionylation. Propionyl-CoA arises from multiple metabolic inputs. Exogenously, propionate produced by gut microbiota through dietary fiber fermentation is absorbed by colonocytes and delivered predominantly to the liver via the portal circulation, with a fraction reaching the systemic circulation, and in host tissues, propionate is activated to propionyl-CoA by ACSS2/3 [10]. Endogenously, propionyl-CoA is generated primarily in mitochondria via several catabolic routes, including the breakdown of branched-chain amino acids (BCAA) (especially isoleucine with a lesser contribution from valine) and other amino acids (e.g., threonine, methionine), β-oxidation of odd-chain fatty acids, and cholesterol side-chain oxidation [11,12]. Under physiological conditions, propionyl-CoA is converted to methylmalonyl-CoA by propionyl-CoA carboxylase and subsequently to succinyl-CoA, entering the tricarboxylic acid (TCA) cycle [11].

Propionyl-CoA availability is a key determinant of lysine propionylation, yet acyl-CoA thioesters are strongly compartmentalized between the mitochondria and the nucleo-cytosol. When mitochondrial propionyl-CoA accumulates, propiogenic carbon can be buffered in part as propionyl-L-carnitine, which may facilitate redistribution to extra-mitochondrial compartments where it can be reconverted to propionyl-CoA. Consistent with this compartmentalized view, recent studies have reported a measurable nuclear propionyl-CoA pool and suggest that nuclear propionyl-CoA availability can be supported by localized metabolic inputs within or near the nucleus, such as nuclear BCAA catabolism [6,13,14]. Notably, because detection of endogenous Kpr have historically been method-limited in some protocols [15,16], recent alternative protocols have suggested that the frequency and abundance of Kpr may have been underestimated [7,17]. Moreover, quantitative analysis revealed that propionyl-CoA is relatively enriched in the nucleus, with a ratio close to 1:1 to acetyl-CoA, especially under metabolic stress conditions such as hypoxia, where nuclear propionyl-CoA levels significantly increase [6].

### 1.2. Historical Context and Key Advances in Lysine Propionylation Research

Research on lysine propionylation has undergone important stages from discovery to an in-depth understanding of its mechanisms (Figure 1). In 2007, this modification was first identified as a histone modification catalyzed by p300/CBP [4]. In 2009, the deacylase SIRT1 was found to be the first reported propionylation eraser in eukaryotes, and K292pr of the p53 protein was identified in vivo by mass spectrometry, albeit with an abundance lower than that of the corresponding acetylation modification [18]. In the same year, multiple conserved propionylation sites were identified in mammalian cells [19], as well as on yeast histones H2B, H3, and H4 [20], suggesting its role as an evolutionarily conserved histone marker.

Subsequent studies expanded our understanding of the breadth and recognition mechanisms of propionylation. In 2014, a whole-proteome propionylation mapping analysis of *Thermus thermophilus* revealed that this modification is widespread in metabolism-related proteins and exhibits growth-stage dependence [21]. In 2015, a systematic screening of bromodomains confirmed that propionylation and associated acylation markers can be recognized by most bromodomains [22]. In 2016, a global proteomics study in *E. coli* identified numerous propionylation sites and highlighted that exogenous propionic acid can significantly increase global protein propionylation levels by enhancing the propionyl-CoA pool [23]. In 2017, research confirmed that GCN5, PCAF, p300 and CBP can catalyze propionylation at the H3K14 site in vitro, and linked propionyl-CoA-mediated chromatin modification with the recognition function of the BAF/PBAF chromatin remodeling complex, establishing a direct link between metabolism and histone propionylation regulation [24]. H4K16 modification is important in regulating chromatin structure and gene transcription. In 2018, an in vitro study demonstrated that MYST family histone acetyltransferases generally possess strong propionyltransferase activity, with MOF targeting sites including H4K16 [25], which was subsequently validated in vivo, where depletion of MOF resulted in the near-complete loss of the H4K16pr mark [26].

In 2019, proteome-wide surveys in *Trichophyton rubrum* profiled lysine propionylation and crotonylation across conidial and mycelial stages, revealing stage-associated acylation landscapes and enrichment of modified proteins in core metabolic and cellular processes, supporting broad involvement of these marks in fungal physiology [27,28]. Kpr may also play an important role in photosynthesis and metabolism of cyanobacteria [29]. Between 2020 and 2023, studies using metabolic intervention models closely linked histone and non-histone propionylation with various pathological processes such as energy metabolism, neurodevelopmental disorders, cancer, and metabolic diseases [6,30,31,32,33]. Since 2023, advances in detection technologies and integrative analyses have moved Kpr research beyond descriptive chromatin marking to proteome-wide, site-resolved functional studies, supporting that Kpr is not merely an extension of Kac but can play determinant roles in transcriptional regulation, protein homeostasis, and disease-relevant regulatory circuits in cancer, cardiovascular disease, and other conditions [8,9,14,33,34,35,36,37,38,39].

Overall, propionylation is a metabolically responsive mark that can display context-dependent, non-redundant regulatory effects at a genome-wide scale, with growing evidence linking it to diverse diseases. This review highlights recent advances in lysine propionylation, emphasizing the metabolic sources of propionyl-CoA, the writer/eraser/reader machinery, molecular mechanisms, and functional roles of histone and non-histone Kpr in physiology and disease.

## 2. Mechanisms of Lysine Propionylation Regulation

Kpr is a metabolically sensitive regulatory layer acting on both histone and non-histone proteins. Its functional output is shaped by propionyl-CoA availability (including compartmentalized pools), writer/eraser activities, and competition among short-chain acyl marks (especially with Kac and Kbu) at shared or nearby lysine residues (Figure 2). In addition to enzyme-catalyzed propionyl transfer, non-enzymatic lysine propionylation has also been observed under high local propionyl-CoA concentrations [40].

### 2.1. The Writers of Kpr

Research on writing enzymes of Kpr has largely focused on canonical lysine acyltransferases (KATs), whose active sites can accommodate short-chain acyl-CoA donors. Early work established that p300/CBP can catalyze histone propionylation and butyrylation, including on histone H4K5, H4K8 and H4K12, and that CBP can also undergo auto-propionylation, consistent with the existence of a permissive acyl-CoA binding pocket [4]. This catalytic activity also extends to non-histone substrates, such as the transcription factor p53 in experimental systems [4]. Structural analyses further support how p300 can engage acyl-CoA variants. The propionyl group can occupy the acyl/lysine channel region and impose steric constraints that require substrate-dependent rearrangements during catalysis [41]. Functionally, p300/CBP-dependent propionylation has been reported at HIF-1α, where the mark is linked to HIF-1α stability and transcription output of target genes [42]. A recent study further reported that CBP can catalyze the propionylation of H3K18 in vitro and in vivo, and identified Tyr1503 in the catalytic core as a key residue required for robust propionyltransferase activity [43].

Members of the MYST family represent another important class of Kpr writers. MOF (KAT8) has been supported as an efficient propionyltransferase by combined evidence from enzymology, proteomic tracing, and high-resolution structural studies. Val314 and Pro349 in its structure are key sites for specific recognition of propionyl-CoA. The core catalytic target of MOF is H4K16, but proteomic data show that its overexpression also leads to propionylation of multiple histone sites such as H4K12, H2AK5, and H2AK9, as well as other broad-spectrum non-histone proteins including MOF itself and p53 [25].

The KAT6 family (MOZ/KAT6A and MORF/KAT6B) is a key writing enzyme for H3K23pr. They require a complex with the scaffold protein BRPF1 to function; knockout of BRPF1 leads to the near-complete disappearance of H3K23pr signaling in mouse embryonic fibroblasts and embryos, while having no significant effect on acylation at other sites, demonstrating the specificity of this modification [37,44]. A recent preprint using pharmacological and genetic interventions further supports that, under specific pathological conditions, H3K23pr is mainly driven by the MYST family, particularly KAT6A and KAT7, while the p300/CBP or GCN5/PCAF pathways have little effect on it [13].

Other acyltransferases have also been shown to participate in the propionylation network. GCN5 (KAT2A) and PCAF (KAT2B) have shown propionyltransferase activity for H3K14 in vitro [45], and p300 can also utilize propionyl-CoA to generate H3K14pr and affect transcription [24]. HBO1 (KAT7) has been shown to catalyze propionylation, butyrylation, and crotonylation of histones both in vivo and in vitro, and is dependent on JADE or BRPF family scaffold proteins. It is a key enzyme for various short-chain acylations at the H3K14 site in cells, and its deficiency leads to a significant decrease in global histone propionylation levels [46].

Furthermore, studies in prokaryotes have provided early models for the reversibility and physiological function of propionylation. Using PrpE as a model in bacteria for the first time, it was revealed that Kpr is a reversible, NAD^+^-dependent metabolic regulatory switch, written by Pat family enzymes and erased by CobB family deacylases, which can translate intracellular propionyl-CoA metabolic stress into precise regulation of enzyme activity [47]. Overexpression of the GNAT family acetyltransferase PatZ in *E. coli* can increase global propionylation levels, suggesting that it has the potential to function as a broad-spectrum propionyltransferase [23]. However, the specificity of modification is not ubiquitous. In cyanobacteria, the deletion of cGNAT2 significantly affects crotonylation but has no effect on the total propionylation level, reflecting the selectivity of enzymes for different acyl donors [48]. While p300 and CBP have been shown to catalyze almost all types of lysine acylations, including propionylation, butyrylation, 2-hydroxylisobutyrylation, β-hydroxybutyrylation, crotonylation and succinylation [41], the GNAT family KATs GCN5 and PCAF have been shown to catalyze propionylation efficiently, but are poorly active for butyrylation and crotonylation [49,50].

### 2.2. The Erasers of Kpr

Removal of Kpr is mediated predominantly by the NAD^+^-dependent sirtuins and in certain contexts, by the classic histone deacetylases (HDACs). Together, these enzymes enable reversible and metabolically coupled control of Kpr.

#### 2.2.1. Sirtuin Family

Sirtuins display broad deacylase activity toward short-chain lysine acylations, including Kpr. Early evidence for reversible propionylation came from prokaryotic systems: the sirtuin-like deacylase CobB in *E. coli* was identified as a key depropionylase, and CobB deficiency increased global Kpr levels [23].

In mammalian cells, multiple sirtuins have been implicated in substrate- and compartment-specific control of propionylation. For example, SIRT1 can remove propionylation at the K709 site of HIF-1α under hypoxic conditions, promoting proteasomal turnover [42]. In mitochondria, SIRT3 regulates the propionylation status of SOD2 at the K132 site [30]. SIRT6 has been reported to interact with the viral protein vIRF1 and reduce its propionylation level, functioning as a depropionylation “brake” in this context [51]. A comparative enzymology study further suggested that SIRT7 exhibits high catalytic efficiency and NAD^+^ affinity toward an H3K18 propionylated peptide substrate [52].

#### 2.2.2. Class I HDAC Family

In addition to sirtuins, classical zinc-dependent HDACs can also remove propionylation. HDAC3 has been identified as an efficient deacylase for Kpr, alongside activity toward Kac and Kbu [53]. A recent preprint’s functional studies further indicate that under BCAA starvation, depropionylation of histone sites such as H3K23, H3K18, and H4K16 is largely mediated by class I HDACs (especially HDAC3), and selective inhibition of HDAC3 increases propionylation levels at these sites [13]. Collectively, these observations place Kpr within the canonical dynamic chromatin regulation framework governed by class I HDAC activity.

### 2.3. The Readers of Kpr

**Figure 3:**
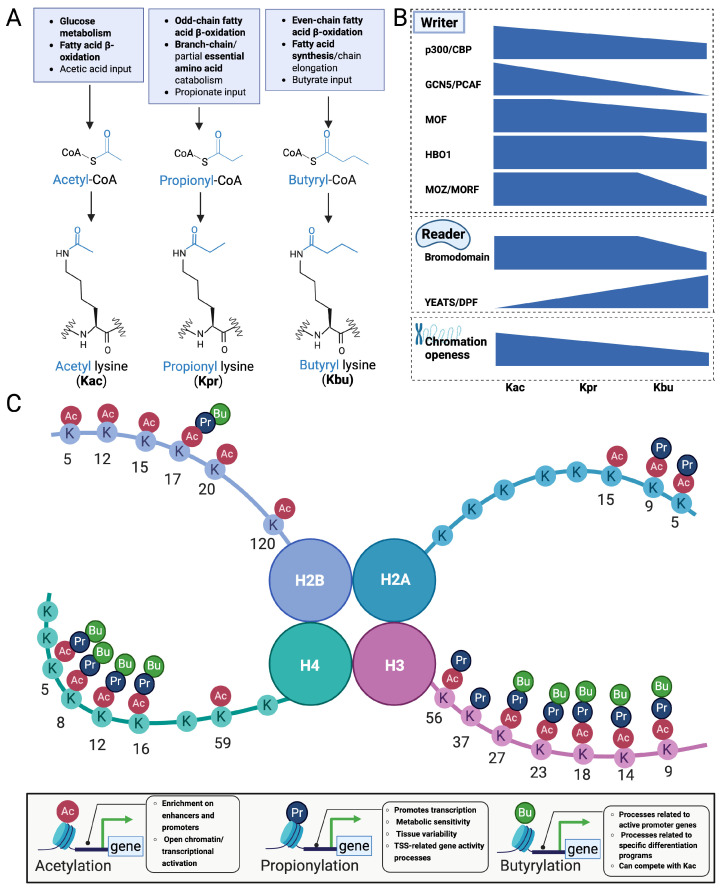
Comparative writer/reader specificity and genomic functions of Kpr, in comparison with Kac and Kbu. (**A**) Schematic overview linking cellular metabolism to three short-chain lysine acylations, showing major metabolic routes that generate acetyl-CoA, propionyl-CoA, and butyryl-CoA (e.g., glucose/acetate-driven acetyl-CoA; odd-chain fatty acid β-oxidation and branched-chain/other propiogenic substrates contributing to propionyl-CoA; even-chain fatty acid β-oxidation/elongation and butyrate input contributing to butyryl-CoA). These acyl-CoA thioesters serve as direct acyl donors for lysine acylation. (**B**) Conceptual depiction that writers (KATs/acyltransferases), readers (acyl-lysine–binding domains), and erasers (HDACs/deacylases) can exhibit differential preferences across acyl marks, shaping mark abundance and signaling. Note that the height of the blue bands indicates relative trends of strength (e.g., enzymatic activity, binding affinity, or functional effect), rather than a strict linear relationship. (**C**) Nucleosome schematic highlighting representative lysine residues on histone tails that can carry acetyl (Ac), propionyl (Pr), or butyryl (Bu) modifications, and simplified functional summary: acetylation is broadly associated with enhancer/promoter enrichment and open chromatin/transcriptional activation; propionylation and butyrylation can overlap with acetylation at shared or nearby lysine residues, yet display context-dependent distributions and transcriptional programs, consistent with metabolite sensitivity, tissue variability, and potential competition among short-chain acyl marks. Created in BioRender. Z.L. (2026) https://BioRender.com.

The downstream effects of acylation depend on reader domains that preferentially bind acyl-lysine marks and recruit effector complexes. Many conserved acyl-lysine recognition modules that bind short-chain lysine acylations also show binding capacity for Kpr, although with diverse binding affinities when compared with Kac and Kbu (Figure 3A,B).

Bromodomain represents the best-characterized acyl-lysine reader family. Kpr can be recognized by multiple bromodomain-containing proteins, including BRD4 [54], BRD9, CECR2, and TAF1 [22]. Subsequent studies provided direct evidence that Kpr can be read by chromatin effector machinery overlapping with that of Kac. Using unbiased peptide pull-down proteomics, H3K14pr and H3K14ac were found to recruit highly similar protein complexes, with multiple subunits of the BAF/PBAF (SWI/SNF) remodelers among the most enriched interactors. Biochemical assays further confirmed that the second bromodomain of PBRM1 (a PBAF subunit) directly binds H3K14pr and H3K14ac peptides with comparable affinity [24].

Beyond bromodomains, DPF and YEATS domains are additional Kpr readers. The DPF domain is known to accommodate multiple H3K14 acyl marks. DPF domains can discriminate among H3K14 acyl states; MOZ-DPF binds H3K14pr and H3K14bu more strongly than H3K14ac (approximately 2.1- and 2.3-fold higher affinity, respectively), with the highest affinity observed for H3K14cr [55]. Direct binding evidence for Kpr has been obtained for the YEATS domain of YEATS2. ITC measurements using H3K27 peptides showed that YEATS2 binds K27pr (*K*_D_ ≈ 148.4 μM) more strongly than K27ac (*K*_D_ ≈ 226.2 μM), with K27bu (*K*_D_ ≈ 123.6 μM) displaying slightly higher affinity than K27pr in the same assay [56] (Figure 3B).

Finally, scaffold proteins can sharpen site selectivity by assembling writer complexes and positioning them on chromatin. BRPF1 acts as an essential scaffold for KAT6A/KAT6B (MOZ/MORF) complexes and is required for robust H3 propionylation at H3K23, with genetic disruption of the BRPF1–KAT6 axis leading to markedly reduced H3K23pr and links to neurodevelopmental disorders and cancer [44]. Mechanistically, BRPF-family scaffolds contain reader modules (including DPF) capable of engaging acylated H3 tails, providing a plausible molecular basis for how scaffolding can coordinate chromatin recruitment and enhance site-selective writing within these complexes [45,55]. Table 1 summarizes key comparative features and representative studies.

### 2.4. Multi-Level Comparison and Dynamic Interaction Between Kpr and Kac

#### 2.4.1. Structural Differences and Recognition Specificity

Although Kpr and Kac are closely related chemically, the additional methylene group in the propionyl moiety increases steric bulk and hydrophobicity relative to acetylation [7]. This difference may lead to changes in the binding characteristics of Kpr with interacting proteins and specific readers, resulting in different transcriptional regulatory outputs. Indeed, multiple reader domains display mark-dependent affinity differences between Kpr and Kac/Kbu (Figure 3B). For instance, in liver cancer tissues, the BET family protein BRD4 has a lower affinity for Kpr than for Kac, while the DPF2 and YEATS domains both have a higher affinity for Kpr than for Kac [57]. These differential binding affinities suggest that Kpr and Kac are preferentially recognized by different reader proteins, resulting in functional specialization despite partial overlap in modification sites (Figure 3B).

#### 2.4.2. Overlap and Independence of Modification Sites

Whole-proteome mapping indicates that Kpr and Kac sites overlap partially. In one dataset, ~19.5% of Kpr sites overlapped with Kac sites, whereas ~35% of Kac sites overlapped with Kpr sites [21]. This asymmetry, while also related to technical challenges in comprehensively detecting these acyl marks, implies that Kpr may not simply be a direct substitute for Kac at the same residues and that the two marks may be differentially regulated across proteins and sites. This partial independence can become more pronounced under defined metabolic perturbations. In a recent preprint, under isoleucine or valine deprivation, histone H3K23pr, H3K18pr and H4K16pr reportedly decreased substantially, whereas H3K9pr and H3K14pr, as well as acetylation at all the corresponding sites, did not change [13]. Propionate stimulation has been reported to preferentially elevate H3K23pr rather than H3K23ac in certain settings [44], illustrating that shifts in substrate availability can bias acylation outcomes at specific loci. While further improvements in high-throughput detection of Kpr remain important, collectively these evidences support a model in which propionylation can be regulated as a partially independent layer rather than a simple “replacement” of acetylation [21].

#### 2.4.3. Selective Catalysis and Removal by Enzyme Systems

Kpr and Kac share many substrate proteins and overlapping enzyme systems, but writers and erasers can display distinct kinetic parameters toward different acyl donors and acyl-lysine substrates (Figure 3B). At the writing level, p300 catalyzes propionylation of histone H3 peptides at a rate approximately three times slower than acetylation [41]. Similarly, in vitro studies of KAT2A/GCN5 have shown lower propionylation yields than acetylation at H3K9 sites (4% vs. 30%) [58]. However, substrate discrimination seems not to be universal. In one study, an HBO1-BRPF2 module showed comparable binding affinity for acetyl-CoA and propionyl-CoA (*K*_D_ 2.02 vs. 2.22 μM), and its catalytic pocket can accommodate multiple acyl-CoA substrates, thus enabling changes in the acyl-CoA substrate pool to be directly reflected in histone modification [46]. At the removal level, sirtuin deacylases can also differentiate among acyl substrates. For instance, Sir2Tm reportedly bound a propionylated p53 peptide 4.4-fold more tightly than an acetylated counterpart (*K*_D_ 0.183 vs. 0.799 μM), consistent with slower product release for the propionyl than the acetyl group (0.141 vs. 0.102 s^−1^) [59]. Together, these observations indicate that cellular steady-state levels of Kpr versus Kac at a given site can be shaped by both writer/eraser preferences and by kinetic steps such as product release, not merely by acyl-CoA abundance (Figure 3A,B).

#### 2.4.4. Dynamic Regulation of the Metabolic Substrate Pool

The absolute concentrations and relative ratios of intracellular acyl-CoA thioesters are key metabolic determinants of Kpr versus Kac. In general, acetyl-CoA is more abundant than propionyl-CoA at the whole-tissue level. In whole-liver extracts, acetyl-CoA typically exceeds propionyl-CoA by an order of magnitude (e.g., ~20:1 in fasted rat liver) [18,41]. Importantly, acyl-CoA pools are compartmentalized, and nuclear ratios can differ from whole-cell averages. Quantitative analyses have suggested that propionyl-CoA is comparable with acetyl-CoA in the nucleus, especially under metabolic stress conditions such as hypoxia, where nuclear propionyl-CoA levels significantly increase [6,42]. Bacterial models further illustrate how nutrient inputs can invert global acylation trends. In *E. coli*, high-glucose conditions were associated with decreased Kpr but increased Kac, whereas propionate exposure increased global Kpr and enriched Kpr on proteins involved in propionate metabolism [23]. The same study reported that a subset of lysine residues can carry both Kac and Kpr and that the two marks may show opposing changes under different nutrient conditions, consistent with competition for shared lysine residues and/or enzyme active sites [23]. Given partial overlap in reader compatibility, shifts in the acyl-CoA ratio could provide a mechanism for metabolically tuned remodeling of chromatin acylation sites [24].

#### 2.4.5. Differences and Similarities Between Genomic Occupancy and Functional Output

At the chromatin level, Kpr and Kac can show both coordinated behavior and mark-biased genomic distribution. In *Drosophila*, H2BK17pr and the active transcriptional marker H3K27ac co-localize in most genes, but their rhythmic occupancy patterns are different, suggesting that Kpr is not solely a passive byproduct of generic transcriptional activity [34]. In addition, genome-wide profiling after propionate treatment indicated that many differential sites were driven primarily by H3K18pr rather than H3K18ac, with higher average signal at those responsive loci [8]. Collectively, these findings argue that short-chain acylations are not merely superimposed on acetylated active regions but can exhibit distinguishable targeting and responsiveness.

Functionally, several studies link propionylation changes to cellular state transitions. Propionate treatment has been associated with reduced total protein levels in multiple cell models, suggesting that increased Kpr may be associated with a protein-reducing cellular state [34]. In metabolic perturbation settings, multiple H4K16 acylations (Kac/Kpr/Kbu) can change in a coordinated direction and co-occur on nucleosomes, supporting a model in which combined acylations weaken internucleosomal interactions and promote chromatin accessibility to sustain transcriptional responsiveness [9]. This concerted acylation framework emphasizes that chromatin outputs may reflect integrated contributions from unique marks rather than a single modification acting in isolation. In another genome-scale study, it was shown that Kpr is preferentially associated with cellular processes such as growth, differentiation, ion transport, and stress response [8], thus also suggesting distinct roles of Kpr (Figure 3C).

Overall, Kpr and Kac are chemically related but differ in steric bulk, hydrophobicity, and compartmentalized acyl-CoA availability. These features shape reader selectivity, writer and eraser kinetics, and site-level overlap, enabling condition-dependent decoupling and reciprocal tuning during metabolic shifts. Evidence from proteomics and chromatin profiling further supports both shared and mark-biased genomic targeting and functional outputs (Figure 3). Table 1 summarizes key comparative features and representative studies.

## 3. Functional Regulatory Mechanism of Propionylation

### 3.1. Gene Regulation and Chromatin Remodeling

H4K8ac has been reported to recruit the SWI/SNF chromatin remodeling complex, raising the possibility that H4K8pr may tune chromatin structure and transcriptional output by reshaping the spectrum and/or affinity of interacting partners at this site. More broadly, short-chain acyl marks (including Kpr) can display localized enrichment at high-turnover regulatory regions such as transcription start sites (TSSs) and may compete with acetylation at shared or nearby lysine residues, enabling fine control of initiation-associated chromatin states and gene expression programs under changing metabolite supply [41].

At the mechanistic level, reader-mediated targeting provides a plausible route for site- and locus-selective actions of Kpr. The DPF domain in MORF-associated factors is known to engage H3K14 and help position MORF-associated acetyltransferase activity on chromatin, potentially facilitating local expansion and promoting transcriptionally permissive chromatin domains [45]. Simultaneously, H3K14pr can be read by bromodomain-containing subunits of the SWI/SNF complex, thereby translating changes in the intracellular metabolic substrate pool into specific outputs of chromatin remodeling and gene expression [24].

Propionylation modifications are widely involved in specific gene regulatory programs. In the fasted livers of mice, the levels of propionylation and butyrylation at histone H3K14 are selectively increased, and these modifications are significantly enriched in the regulatory regions of lipid metabolism-related genes, demonstrating pathway selectivity in metabolic adaptation [24]. Under exogenous propionate stimulation, H3K23pr was elevated in the core enhancer, distal, and proximal regulatory regions of the *MYOD* gene, indicating that increased propionylation is not limited to a single regulatory element but can extend to a wider range of genomic regions [32]. A metabolic tracking study, by examining four endogenous propionylated peptides, found increased isotope incorporation in H2AK5pr, H4K16pr, and H3K23pr, while H2BK14pr did not exhibit this phenomenon. This supports the idea that propionyl groups produced by isoleucine catabolism can preferentially contribute to specific histone propionylation sites [6]. The dynamic changes in propionylation modifications are closely related to biological rhythms. A study using a *Drosophila* model identified H2BK17pr as a highly predictive site with significant temporal dynamics through quantitative propionylomics, and confirmed the specificity of this modification using a site-specific mutation model. Further H2BK17pr ChIP-seq analyses showed that this modification is highly enriched in the promoter region near the transcription start site and, under circadian clock gating, translates time-limited feeding signals into rhythmic enhancement of chromatin occupancy, thereby mediating rhythmic expression of downstream proteasome genes [34]. In a cardiac stress-load model, H3K23pr is not a genome-wide alteration, but rather specifically enriched in the promoter regions of workload-related genes, and occurs in conjunction with the upregulation of extracellular matrix genes and proliferation markers [35]. Furthermore, propionic acid produced by gut microbial fermentation can affect the acylation metabolic pool of host intestinal epithelial cells, dynamically regulating sites such as H3K27pr and H3K9pr. These modifications are linked to regulation of epithelial gene expression [60]. At the metabolic level, specific pathways directly provide precursors for histone propionylation. The oxidative catabolism of BCAA can generate propionyl-CoA, which in turn supports propionylation at histone H3K23. Blocking further metabolic consumption of propionyl-CoA leads to its intracellular accumulation and selectively upregulates specific sites such as H3K23pr and H3K18pr, revealing metabolic flux-dependent regulation of site-selective histone propionylation [14]. A study found that the genome-wide distribution of H3K18pr/bu and H4K12pr/bu induced by short-chain fatty acids (SCFA) differs significantly from the classic acetylation markers H3K18ac/H4K12ac. These short-chain acylation modifications are not only enriched near transcription start sites but also extend more extensively downstream of the TSS and to some distant regulatory regions, showing a high overall correlation with open chromatin states and corresponding to gene activation in pathways such as growth, differentiation, ion transport, and stress response [8].

Modifications such as propionylation also directly affect the higher-order structure of chromatin. Studies have found that H4K16pr and H4K16bu, along with H4K16ac, are enriched in actively expressed genes and open chromatin regions, supporting their association with chromatin activity [9]. Physically, DNA origami force spectroscopy experiments showed that various acylation modifications at the H4K16 position can push nucleosome conformations to a larger opening angle, suggesting that they can weaken interactions between nucleosomes. Acetylation has the strongest effect, followed by propionylation and butyrylation. Single-molecule FRET analysis further confirmed that the proportion of closely stacked conformations decreased and the proportion of open conformations increased in chromatin fibers containing H4K16 acylation modifications, concluding that various acylation forms of H4K16 can promote a more open chromatin fiber state, with varying contributions from H4K16ac, H4K16pr, and H4K16bu [8].

### 3.2. Regulation of Enzyme Activity and Metabolic Pathways

Propionylation can directly regulate the activity and function of non-histone enzymes. In studies of the mitochondrial antioxidant enzyme SOD2, a propionylation-mimicking mutation at lysine 132 leads to decreased enzyme activity, increased reactive oxygen species levels, and reduced cell viability, whereas a mutation blocking propionylation at this site maintains SOD2 activity under metabolic stress. Global propionylomics analysis further revealed extensive propionylation enrichment in proteins related to oxidative phosphorylation and the tricarboxylic acid cycle [30]. Consistently, metabolic conditions such as a high-fat diet can promote SOD2 K132 propionylation through propionyl-CoA accumulation and Sirt3 downregulation, linking metabolic imbalance to mitochondrial oxidative stress and contributing to intestinal injury [30]. Propionylation is also a key mechanism for viral regulation of host immunity. The Kaposi’s sarcoma-associated herpesvirus protein vIRF1 effectively competes for the host transcriptional coactivator p300/CBP and inhibits the STING-TBK1-IRF3 signaling axis through propionylation at specific lysine sites, thereby blocking the host antiviral innate immune response; mutations at these sites significantly weaken its immunosuppressive function [51].

Propionylation is widely involved in the regulation of metabolic pathways. In bacteria, when propionate is used as a carbon source, key enzymes in assimilation and degradation pathways, such as propionyl-CoA synthase PrpE and PrpC in the methylcitrate cycle, exhibit multi-site specific propionylation. These modifications are minimal under normal culture conditions, suggesting that protein propionylation can act as an accompanying regulatory mechanism when propionate metabolism is activated. Site mapping further indicated that K80 and K234 of PrpC are often located near the substrate-binding pocket or active site. Moreover, functional mutagenesis of the conserved K609 site in ACS showed reduced enzyme activity, indicating that propionylation may directly regulate the activity and metabolic flux of key metabolic enzymes [23]. In mammalian systems, changes in metabolic state can also trigger specific histone acetylation responses. Vai et al. reported that histone propionylation was relatively higher in spleen and heart tissues of mice, whereas it was lower in the liver and kidney [7].

### 3.3. Protein Stability and Degradation

The regulatory function of propionylation extends beyond chromatin, encompassing aspects of cellular metabolism and protein homeostasis. Global protein propionylomics analysis revealed that modified substrates are significantly enriched in networks related to ribosomes, glycolysis/pyruvate metabolism, and aminoacyl-tRNA synthesis, suggesting that propionylation may alter bacterial resource allocation under different carbon source environments by modulating translational machinery and key proteins in central carbon metabolism [23].

**Figure 4:**
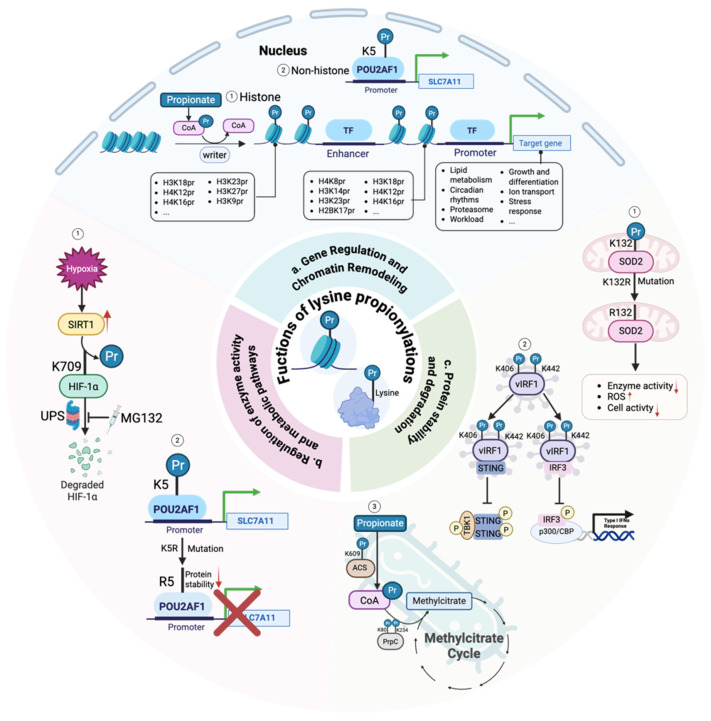
Metabolic and epigenetic functions of Kpr. Propionate-derived propionyl-CoA supports lysine propionylation on histones and non-histone proteins in the nucleus and mitochondria. Histone propionylation at enhancers and promoters modulates chromatin accessibility and gene expression programs linked to lipid metabolism, circadian rhythm, proliferation, inflammation, senescence, stress responses, and cancer. Propionylation of the transcriptional cofactor POU2AF1 enhances *SLC7A11* transcription, whereas lysine substitution diminishes this activity. Propionylation of chromatin-associated repair factors tunes DNA damage responses. Sirtuin-dependent depropionylation influences protein stability through ubiquitin proteasome turnover, exemplified by HIF-1α control. In mitochondria, SOD2 propionylation reduces antioxidant function, increases ROS, and affects cell fate. Propionate catabolism links methylmalonate and methylcitrate pathways, providing a metabolic conduit to nuclear signaling and genome maintenance. Created in BioRender. Z.L. (2026) https://BioRender.com.

Propionylation is also an important mechanism regulating protein stability. Propionate can induce upregulation of the E3 ubiquitin ligase HECTD2. HECTD2 catalyzes polyubiquitination of the histone methyltransferase EHMT2 and promotes its proteasomal degradation, revealing a pathway by which microbial metabolites influence host epigenetics through regulation of protein stability [61]. Under hypoxic stress, overexpression of the deacylase SIRT1 reduces propionylation at K709 of HIF-1α and promotes its degradation. This process can be blocked by the proteasome inhibitor MG132, suggesting that depropionylation may render target proteins more susceptible to ubiquitin-proteasome turnover [42]. Propionylation also plays a regulatory role in the proteasome pathway itself. Propionylation of histone H2B K17/K23 can enhance global protein degradation capacity by regulating transcription of ubiquitin-proteasome system-related genes, thereby influencing tissue-level protein homeostasis [34]. Furthermore, propionylation at the K5 site of the transcription cofactor POU2AF1 has been shown to be crucial for its protein stability; mutations at the K5 site reduce POU2AF1 stability and weaken its ability to bind and activate promoters of downstream target genes such as *SLC7A11*, highlighting the role of site-specific propionylation in maintaining transcription factor/cofactor function [62] (Figure 4).

## 4. Propionylation in Diseases: Associations, Mechanistic Insights, and Causal Inference

The role of propionylation in disease can be context-dependent. While numerous studies have reported associations between altered propionylation and pathological states, the extent to which these modifications act as causal drivers, functional contributions, or merely biomarkers of metabolic dysregulation varies and remains to be further elucidated.

### 4.1. Propionylation in Metabolism-Related Diseases

Altered propionyl-CoA metabolism represents a primary driver linking metabolic disorders to dysregulated propionylation. In propionic acidemia, deficiency of propionyl-CoA carboxylase leads to pathological accumulation of propionyl-CoA, which is accompanied by a global increase in protein propionylation, including histones, as observed in patient-derived fibroblasts and mouse models [9,31]. These findings support a direct metabolic-epigenetic coupling in which excess substrate availability drives widespread acylation changes. Beyond rare genetic disorders, similar metabolic shifts may also occur under physiological conditions. For example, human muscle samples from older individuals exhibit elevated propionylcarnitine levels, suggesting age-related alterations in substrate availability and metabolic flux [32], highlighting the importance of functionally dissecting the role of propionylation in aging-related metabolic regulation.

At the functional level, such alterations in acetylation are associated with cellular dysfunction. In animal models, elevated propionyl-CoA reshapes the acylation landscape, with increased H4K16pr and redistribution of other acyl marks, including acetylation and butyrylation, suggesting competitive dynamics among short-chain acyl modifications under metabolic stress [9]. These changes occur in parallel with mitochondrial respiratory defects [31], although a direct causal link between specific propionylation sites and mitochondrial dysfunction remains to be fully established.

Upstream regulatory pathways further modulate this process. For example, loss of the mitochondrial inner membrane protein ACSS3 impairs propionate clearance and promotes obesity-like metabolic dysregulation through mTOR signaling [63], while in nonalcoholic fatty liver disease (NAFLD), RPN11-mediated regulation of ACSS3 enhances propionyl-CoA production and histone propionylation (e.g., H3K14pr, H3K23pr), supporting lipid metabolic programs [36]. These studies suggest that propionyl-CoA metabolism functions as a regulatory node connecting metabolic flux to epigenetic remodeling.

**Figure 5:**
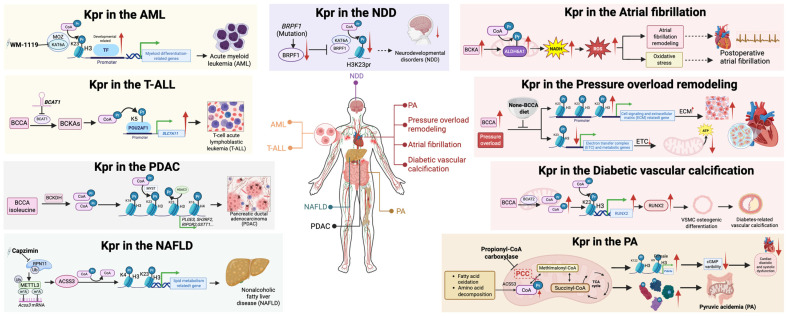
Disease mechanisms and therapeutic targeting of Kpr. This schematic summarizes disease-specific roles of Kpr and corresponding therapeutic strategies across metabolic, cardiovascular, neurological, and malignant disorders. Altered propionyl CoA metabolism and site-selective Kpr can reshape transcriptional programs, protein stability, and stress responses in acute myeloid leukemia, T-cell acute lymphoblastic leukemia, pancreatic ductal adenocarcinoma, nonalcoholic fatty liver disease, neurodevelopmental disorders, atrial fibrillation, pressure overload-induced cardiac remodeling, diabetic vascular calcification, and propionic acidemia. The schematic further highlights intervention approaches including inhibition of propionylation writers, modulation of depropionylating enzymes, metabolic reprogramming of propionyl CoA supply, dietary or amino acid-based strategies, and pharmacological targeting of upstream metabolic nodes. Together, the diagram emphasizes Kpr as a metabolite-sensitive regulatory hub that can contribute to disease progression and may provide actionable entry points for therapeutic intervention. Created in BioRender. Z.L. (2026) https://BioRender.com.

Emerging evidence also indicates that compartmentalized expansion of propionyl-CoA pools can promote H3K23pr deposition at specific genomic regions, such as the MYOD regulatory regions, via p300/CBP and can be accompanied by changes in acetylation. Such an imbalanced acylation landscape may disrupt the epigenetic timing required for myocyte differentiation, leading to impaired differentiation, raising the possibility that propionyl-CoA/Kpr imbalance in aging or metabolic stress contributes to disease development [32] (Figure 5).

### 4.2. Propionylation in Cancer

Increasing evidence indicates that lysine propionylation contributes to tumorigenesis through both histone-dependent transcriptional regulation and non-histone signaling pathways. In hematological malignancies, histone propionylation has been implicated in transcriptional dysregulation driven by oncogenic chromatin modifiers. In MOZ-TIF2-associated acute myeloid leukemia, H3K23pr levels are elevated and decrease in a time- and dose-dependent manner upon treatment with the KAT6 inhibitor WM-1119 [37]. Mechanistically, the MOZ-TIF2 fusion protein is enriched at promoter regions and promotes accumulation of H3K23pr near transcription start sites of highly expressed genes in a KAT6 enzymatic activity-dependent manner, thereby activating developmental transcription factors. In a mouse model of leukemia, the findings links KAT6 enzymatic activity to genome-wide H3K23pr redistribution, transcriptional output, and pharmacological intervention, supporting a rationale for targeted therapy [37]. While this study provides strong evidence that H3K23pr is dynamically regulated and associated with transcriptional activation, the simultaneous decrease in H3K23ac upon KAT6 inhibition makes it difficult to disentangle the specific functional contribution of propionylation from acetylation.

Beyond histones, propionylation of non-histone proteins can also regulate tumor cell phenotypes. In T-cell acute lymphoblastic leukemia (T-ALL), elevated BCAT1 expression and enhanced BCAA metabolism increase propionyl-CoA supply and promote global protein propionylation. Notably, propionylation of the transcriptional coactivator POU2AF1 at K5 upregulates expression of the cystine transporter *SLC7A11*, suppresses ferroptosis, and supports T-ALL proliferation, self-renewal, and bone marrow homing, and is associated with poor prognosis [62].

A recent preprint work has further highlighted a direct link between nuclear metabolic reprogramming and Kpr. In pancreatic ductal adenocarcinoma (PDAC), a nuclear isoleucine-derived propionyl-CoA pathway via BCKDH complex has been proposed, supports local propionyl-CoA generation in the nucleus. This nuclear Ile-Kpr axis drives transcriptional programs associated with lipid metabolism and immune regulation and promotes tumor growth. Genetic disruption of the BCKDH subunit DBT suppresses tumor growth and increases CD3^+^ T-cell infiltration, suggesting that metabolite-driven chromatin propionylation can influence both tumor progression and the immune microenvironment [13].

In hepatocellular carcinoma (HCC), propionyl-CoA levels have been reported to be decreased and negatively correlated with TNM stage and Edmondson grade, suggesting a metabolic association between propionyl-CoA homeostasis and tumor progression [57]. Further work suggests that downregulation of ALDH6A1 reduces propionyl-CoA production and may impact citrate activity and the TCA cycle, thereby affecting HCC proliferation and migration [63,64]. However, whether these metabolic alternations are directly through Kpr remains to be clarified.

### 4.3. Propionylation in Cardiovascular Diseases

Increasing evidence suggests that alterations in propionyl-CoA metabolism and protein propionylation contribute to cardiovascular pathophysiology. In models of propionic acidemia, elevated cardiac propionyl-CoA levels are associated with increased histone acetylation and propionylation, including modifications at the *Pde9a* locus. The enhanced *Pde9a* transcription remodels cGMP signaling, contributing to both systolic and diastolic dysfunction. Sex-dependent differences have also been reported, with female hearts exhibiting greater propionyl-CoA accumulation, higher histone propionylation/acetylation levels, stronger remodeling of genes such as *Pde9a*, and more pronounced diastolic dysfunction. In contrast, increased β-alanine levels in males may buffer propionyl-CoA accumulation, partially mitigating disease severity [33].

Beyond systemic metabolic disorders, local metabolic pathways can also shape cardiac chromatin acylation. In stress-overloaded hearts, nuclear BCAA oxidation has been proposed to supply propionyl-CoA locally, promoting H3K23pr remodeling at promoter regions of extracellular matrix and stress-response-related genes. Dietary BCAA restriction attenuates these H3K23pr changes and reduces cardiac fibrosis and cardiomyopathy progression, supporting a role for metabolite-driven chromatin propionylation in cardiac stress responses [14].

Propionylation of non-histone proteins may further contribute to cardiovascular pathology. In atrial tissue from patients with new-onset atrial fibrillation after coronary artery bypass grafting, ALDH6A1 K113pr enhances enzyme activity of ALDH6A1 by altering NAD^+^ binding, leading to NADH accumulation and mitochondrial oxidative stress, thereby promoting atrial electrical remodeling and susceptibility to atrial fibrillation [65]. Similarly, in diabetes-related vascular calcification, enhanced BCAA catabolism increases BCAT2-mediated propionyl-CoA production and elevates H3K23pr at the promoter of RUNX2, upregulating RUNX2 expression and driving osteogenic transdifferentiation of vascular smooth muscle cells, thereby exacerbating diabetic atherosclerotic calcification. Genetic deletion of BCAT2 reduces propionyl-CoA and H3K23pr levels, suppresses RUNX2 activation, and alleviates vascular calcification [39].

Together, these studies suggest that cardiovascular propionylation can arise from both systemic metabolic imbalance and localized metabolic reprograming, linking propionyl-CoA availability to chromatin remodeling, mitochondrial stress responses, and vascular pathology.

### 4.4. Propionylation in Neurodevelopmental Disorders

Proteomics analyses reveal that lysine propionylation is readily detectable in brain tissue [7]. A mechanistic connection between propionylation and neurodevelopmental disorders has emerged from studies of BRPF-associated syndromic intellectual disability. BRPF1 functions as a scaffold protein for the MYST family acetyltransferases KAT6A and KAT6B, which catalyze both histone H3K23ac and H3K23pr. Syndromic intellectual disability-associated BRPF1 variants impair activation of KAT6A/KAT6B complexes, resulting in reduced H3K23pr deposition. Consistent with this mechanism, reduced H3K23pr levels have been detected in patient-derived cells carrying BRPF1 mutations, providing in vivo evidence that disruption of the BRPF1-KAT6 complex may alter chromatin acylation states and transcriptional programs relevant to neurodevelopment [44]. However, the extent to which H3K23pr exerts functions independent of, or coordinated with, H3K23ac remains to be determined.

### 4.5. Disease Intervention Strategies Targeting Kpr

Therapeutic manipulation of Kpr can be achieved through two complementary routes. One direct strategy is to modulate enzymatic “writers” or “erasers” that install or remove propionyl marks, thereby reshaping site-specific and global Kpr states. Another indirect approach is to alter propionyl-CoA availability through dietary inputs or metabolic pathway interventions, coupling nutrient status to chromatin and proteome propionylation. Table 2 summarizes representative pharmacologic and metabolic interventions, their mechanisms, and disease contexts.

#### 4.5.1. Direct Pharmacological Intervention Based on Targeted Modified Enzymes

Directly targeting Kpr writing and erasing enzymes is a core therapeutic direction. Histone acetyltransferases such as p300/CBP and KAT6 complexes, members of the MYST family, have been confirmed as important propionyl “writer” enzymes. Studies have found that p300/CBP inhibitors C646 and A485 can inhibit propionate-induced increases in histone propionylation or reduce propionylation at specific sites such as H2BK17 [32,34]. Further research in a Drosophila model suggests that the TIP60/MOF inhibitor MG149 can also dose-dependently reduce H2BK17pr [34]. In the context of cancer, structural rearrangements or amplifications of KAT6A or KAT6B are common, and their catalytic domains are usually preserved, suggesting that targeting these aberrantly activated “writer” enzymes may have therapeutic value [44]. Targeting depropionylases is another effective means of restoring modification balance. Histone deacetylase inhibitors, such as VPA, TSA, and SAHA, can increase global protein propionylation levels, including histones, by blocking the removal of propionyl groups [38]. In Kaposi’s sarcoma-associated herpesvirus infection, the viral protein vIRF1 maintains a high propionylation state by inducing degradation of the depropionylase SIRT6, thereby inhibiting the host interferon response; this process can be reversed using the SIRT6-selective activator UBCS039, restoring antiviral immunity [51]. This strategy has been innovatively applied in cell therapy. Studies have confirmed that VPA can be metabolized into propionyl-CoA in CAR-T cells, together with its HDAC inhibitory activity, lead to enrichment of H3K56pr at specific promoter regions, thereby transcriptionally activating gene networks related to cell migration, adhesion, and metabolic adaptation, ultimately enhancing infiltration, persistence, and antitumor efficacy of CAR-T cells in solid tumors [38].

#### 4.5.2. Indirect Intervention Strategies Based on Metabolic Regulation

Regulating intracellular propionyl-CoA availability is a crucial strategy for modulating Kpr. SCFAs such as propionate are not only GPCR ligands or HDAC inhibitors, but can also modulate histone propionylation at specific sites via propionyl-CoA, thus linking dietary fiber fermentation to epigenetic regulation [44]. Studies have confirmed that exogenous propionate treatment dose-dependently increases propionylation at the H2BK17 site, suggesting that feeding rhythms may drive dynamic changes in histone modification by altering propionyl-CoA donor availability.

BCAA metabolism is another key source of propionyl groups. In stress-induced cardiac remodeling, dietary isoleucine alters intranuclear propionyl-CoA availability, regulating H3K23pr to remodel the transcriptional programs in a promoter-specific manner, driving the fibrotic process [35]. The RPN11 inhibitor Capzimin has been proposed as a therapeutic strategy to intervene in the NAFLD propionylation axis [36]. In T-cell acute lymphoblastic leukemia, BCAT1-mediated BCAA degradation is crucial for maintaining propionylation. The use of the BCAT1 inhibitor gabapentin or a low-BCAA diet combined with PD-1 blockade can synergistically inhibit leukemia progression [62].

## 5. Conclusions

Lysine propionylation research has evolved from initial phenomenological descriptions to in-depth exploration of its mechanism as a metabolically driven, reversible regulatory layer. Kpr represents a distinctive post-translational modification that operates at the interface of metabolism and epigenetics [43], exhibiting both shared machinery with acetylation and mark-specific functional properties. The evidence synthesized in this review indicates that Kpr occupies an intermediate position between complete functional independence and total redundancy with the well-characterized Kac. On one hand, Kpr and Kac share common writer and eraser enzymes, exhibit site overlaps, and can co-localize on chromatin—findings that might initially suggest functional redundancy [34,41,58,59]. On the other hand, they display mark-specific reader affinities, distinct kinetics for enzymatic processing, steric differences arising from the additional methylene group in propionylation, and differential responses to metabolic perturbations [6,13,18,41,42,57]. These biochemical and regulatory differences are further supported by recent unbiased omic studies, which indicate that Kpr can exert functions that are not fully interchangeable with Kac. Thus, these seemingly contradictory observations—shared enzymatic machinery alongside distinct functional roles—can be reconciled by viewing Kpr not as a simple substitute for Kac, but as a partially independent, context-dependent regulatory layer. At sites where both marks can occur, their relative abundance is shaped by multiple intersecting factors, including the compartmentalized availability of specific acyl-CoA substrates, the kinetic preferences of writers and erasers toward different acyl chains, and structural constraints imposed by the propionyl moiety itself. This multi-layered regulation enables condition-dependent decoupling, allowing propionylation to serve as a metabolically sensitive modulator that can either reinforce acetylation-mediated outputs or direct distinct functional outcomes depending on cellular state. Thus, while shared enzymatic machinery and chromatin co-localization provide a foundation for coordinated regulation, biochemical, structural, and context differences enable functional specialization. This integrated framework helps resolve the apparent paradox of shared machinery producing distinct functional outcomes and highlights the importance of exploring Kpr as a regulatory modification in its own right, rather than merely as a variant of acetylation.

As a modification highly sensitive to propionyl-CoA abundance, Kpr is regulated by metabolic networks. Alterations in metabolic pathways affect propionyl-CoA production, clearance, and potential intercompartmental transport [6,66], ultimately translating metabolic signals into propionylation at specific histone sites. These marks can then be coupled to transcriptional outputs via reader systems such as bromodomain-containing proteins. The formation mechanism of this modification is context-specific: chromatin modification H3K23pr exhibit high enzymatic site selectivity, primarily catalyzed by lysine acyltransferases. In cellular compartments with high propionyl-CoA concentrations in mitochondria, propionylation of many metabolic enzymes is closely related to metabolite levels and deacylase activity, and may occur non-enzymatically at lower levels [43]. Novel derivatization techniques and optimized mass spectrometry analysis procedures that utilize relabeling reagents to address homogenization effects can further enable the precise detection and quantification of novel modifications such as propionylation and butyrylation [7]. Methodological advancements are also continuously improving: optimizing sample processing and mass spectrometry strategies has improved the detection reliability of endogenous histone propionylation sites such as H3K27pr and H3K9pr in tissues such as the intestine [60]. Moreover, novel detection tools such as the spectroscopic ProCharTS method have been developed, enabling rapid and low-cost assessment of modification levels. However, significant challenges remain in this field. Because histone Kpr is typically low in abundance, densely packed, and coexisting with other acylation modifications, detectability does not equate to quantification. Conventional proteomics workflows face challenges in short peptide analysis, batch-to-batch bias control, and the quantitative stability of low-abundance modifications. Furthermore, ChIP-seq evidence in functional studies is highly dependent on antibody specificity, and the selection of high-quality antibodies targeting specific sites, such as H3K23pr, is limited. This necessitates extreme caution in interpreting the causal relationship between modifications and phenotypes. Overall, compared to the more mature acetylation or ubiquitination studies, Kpr remains relatively underdeveloped in terms of reader protein systems, site-specific functional validation, and the development of druggable targeting tools. It represents a rapidly evolving PTM undergoing mechanistic elucidation.

## 6. Outlook

Future research needs to further explore the functional nature and regulatory logic of lysine propionylation. At the basic science level, the primary challenge is identifying which Kpr sites are regulatory sites with specific biological functions, rather than simply overflow byproducts driven by metabolite concentration fluctuations, and this requires developing more precise methods to quantify the functional stoichiometry at endogenous levels. Simultaneously, the complete dynamic erasure mechanism remains unclear, particularly how class I histone deacetylases (HDACs) and sirtuins collaborate in different cellular compartments to perform depropionylation. In terms of molecular recognition, it is necessary to explore the chromatin environment in which the multivalent binding of reader proteins can transform the previously weak binding force of a single Kpr label into a biologically meaningful stable interaction. In translational medicine, a key proposition is whether it is possible to construct disease-specific therapeutic windows by targeting specific upstream regulatory axes (e.g., the BRPF1-KAT6 complex), rather than globally inhibiting acetyltransferase activity, to improve efficacy and reduce toxicity. Furthermore, current research lacks evidence of propionylation-specific pathogenicity in respiratory and most neurological diseases, making these areas valuable research directions for integrating metabolomics, acylation proteomics, and chromatin analysis techniques.

## Figures and Tables

**Table 1:** Comparative framework for Kpr versus Kac across recognition, regulation, and functional outputs.

Level	Kpr	Kac	Key Readouts	Source
Chemical properties	One extra methylene; bulkier, more hydrophobic	Shorter acyl; smaller, less hydrophobic	Physicochemical shift enables differential recognition	[1]
Reader specificity	Higher affinity for YEATS domains	Higher affinity for BRD4 BET bromodomain	BRD4 prefers Kac; YEATS prefers Kpr	[57]
Co-enriched modules	Ribosome proteins co-enriched with Kac, succinylation	Ribosome proteins co-enriched with Kpr, succinylation	Suggests dense cross-regulatory acylation hotspots	[28]
Site overlap	Most Kpr sites lack Kac	Most Kac sites lack Kpr	Kpr ∩ Kac 19.5%; Kac ∩ Kpr 35%. Note: potential technical limitations	[21]
Nutrient-stress independence	Ile/Val deprivation lowers H3K23pr, H3K18pr, H4K16pr	Corresponding acetylation unchanged at those sites	Decoupling supports independent regulation	[13]
Substrate-driven bias	Propionate preferentially increases H3K23pr	H3K23ac not preferentially increased	Substrate pool shifts site-specific marking	[44]
Writer kinetics and efficiency	p300 writes Kpr about threefold slower	p300 writes Kac faster	KAT2A: H3K9pr 4% vs. H3K9ac 30%	[41,58]
Writer donor permissiveness	HBO1–BRPF2 binds propionyl-CoA similarly to acetyl-CoA	HBO1–BRPF2 binds acetyl-CoA similarly to propionyl-CoA	Permissive pocket couples donor ratios to marks	[46]
Eraser selectivity	Sir2Tm binds Kpr stronger; turnover slightly lower	Sir2Tm binds Kac weaker; turnover slightly higher	Product release kinetics shape steady-state levels	[59]
Acyl-CoA availability	Lower in whole liver; in nucleus can approach acetyl-CoA	Higher in whole liver; nuclear dominance; reduced under stress	Fasted liver 20:1; nuclear near 1:1	[6,18,41,42]
Global balance and competition	Propionate raises global Kpr; enriches propionate-metabolism pathways	Often inversely changes at dual-modified sites	119 sites carry both; opposite nutrient responses	[23]
Ratio-based chromatin tuning	Shared readers allow tuning via propionyl-CoA fraction	Shared readers allow tuning via acetyl-CoA abundance	Acyl-CoA ratio may modulate transcription	[24]
Genomic occupancy	Propionate shifts differential peaks toward H3K18pr	H3K18ac contributes fewer differential peaks	Co-localizes with H3K27ac; rhythmic patterns differ	[8,34]
Functional outputs and synergy	Propionate-associated Kpr correlates with reduced total protein	Co-varies with Kpr and Kbu at H4K16	H4K16 Kac/Kpr/Kbu co-exist; support transcriptional robustness	[9,34]

**Table 2:** Direct and indirect intervention strategies targeting Kpr across diseases.

Drug Name	Intervention Drug	Mode of Action	Direct/Indirect	Disease Type	Source
C646	C646	p300/CBP inhibition	Direct	Experimental model, histone Kpr regulation	[32,34]
A485	A485	p300/CBP inhibition	Direct	Model context, histone Kpr regulation	[32,34]
MG149	MG149	TIP60/MOF inhibition	Direct	Drosophila model, histone Kpr regulation	[34]
VPA	Valproic acid	HDAC inhibition; converted to propionyl-CoA	Direct	Solid-tumor CAR-T enhancement; global Kpr modulation	[38]
TSA	Trichostatin A	HDAC inhibition	Direct	Global Kpr modulation	[38]
SAHA	SAHA	HDAC inhibition	Direct	Global Kpr modulation	[38]
UBCS039	UBCS039	SIRT6 activation	Direct	KSHV infection, antiviral immunity	[51]
Propionate	Propionate	Increases propionyl-CoA supply	Indirect	Diet-linked epigenetic regulation	[32,44]
Dietary fiber fermentation	Dietary fiber	Raises propionyl-CoA via fermentation	Indirect	Diet-linked epigenetic regulation	[44]
Dietary isoleucine	Isoleucine	Alters nuclear propionyl-CoA via BCAA catabolism	Indirect	Stress-induced cardiac remodeling	[35]
Capzimin	Capzimin	RPN11 inhibition	Indirect	NAFLD	[36]
Gabapentin	Gabapentin	BCAT1-pathway suppression	Indirect	T-ALL	[62]
Low-BCAA diet + PD-1 blockade	Low-BCAA diet + PD-1 blockade	Limits BCAA-derived propionyl groups; immunotherapy synergy	Indirect	T-ALL	[62]

## Data Availability

The data presented in this study are available on request from the corresponding author due to data are not publicly available as they form part of ongoing or planned analyses.

## Abbreviations

The following abbreviations are used in this manuscript:

ACSS	Short-chain member of the acyl-CoA synthase family
BAF/PBAF	BRG1/BRM-related factor/polybrominated BAF complex
BCAA	Branched-chain amino acids
BCAT1/2	Branched-chain amino acid transaminases 1/2
BCKDH	Branched-chain ketoate dehydrogenase complex
BRD4	Bromodomain protein 4
BRPF1	Bromodomain and PHD finger domain protein 1
CAR-T	Chimeric antigen receptor T cell
CBP	CREB-binding protein
ChIP-seq	Chromatin immunoprecipitation sequencing
CrAT	Carnitine O-acetyltransferase
DPF	Double PHD Hepatic domain
EHMT2	Euchromatin histone lysine methyltransferase 2
FRET	Fluorescence resonance energy transfer
GCN5	Universally controlled non-repressor protein 5
GPCR	G protein-coupled receptor
HBO1	Histone acetyltransferase bound to ORC1
HCC	Hepatocellular carcinoma
HDACs	Histone deacetylases
HECTD2	HECT domain E3 ubiquitin ligase 2
HIF-1α	Hypoxia-inducible factor 1α
Kac	Lysine acetylation
KAT	Lysine acetyltransferase
Kpr	Lysine propionylation
MOF	Male-deficient factor, also called KAT8
mTOR	Mammalian target of rapamycin
MYOD	Myogenic differentiation protein
NAD^+^	Nicotinamide adenine dinucleotide
NAFLD	Nonalcoholic fatty liver disease
PCAF	p300/CBP-related factor
PBRM1	Polybromin 1
PCCA	Propionyl-CoA carboxylase subunit α
PCCB	Propionyl-CoA carboxylase subunit β
PDAC	Pancreatic ductal adenocarcinoma
PDE9A	Phosphodiesterase 9A
Pr-CoA	Propionyl-CoA
ProCharTS	Time-resolved spectrophotometry for propionylation characterization
PTMs	Post-translational modifications of proteins
ROS	Reactive oxygen species
RUNX2	Runt-related transcription factor 2
SAHA	S-saturated aniline hydroxamic acid
SIRT1/3/6/7	Silent signaling regulator 1/3/6/7
SOD2	Superoxide dismutase 2
STING-TBK1-IRF3	Interferon gene stimulating protein-tank-binding kinase 1-interferon regulatory factor 3
T-ALL	T-cell acute lymphoblastic leukemia
TCA	Tricarboxylic acid cycle
TSA	Trichostatin A
VPA	Valproic acid
YEATS	Yaf9, ENL, AF9, Taf14, Sas5 domains
